# Renalase Deficiency in Heart Failure Model of Rats—A Potential Mechanism Underlying Circulating Norepinephrine Accumulation

**DOI:** 10.1371/journal.pone.0014633

**Published:** 2011-01-31

**Authors:** Rong Gu, Wen Lu, Jun Xie, Jian Bai, Biao Xu

**Affiliations:** Department of Cardiology, Affiliated Drum Tower Hospital, Nanjing University Medical School, Nanjing, China; Harvard Medical School, United States of America

## Abstract

**Background:**

Sympathetic overactivity and catecholamine accumulation are important characteristic findings in heart failure, which contribute to its pathophysiology. Here, we identify a potential mechanism underlying norepinephrine accumulation in a rat model of heart failure.

**Methodology/Principal Findings:**

Initially, we constructed a rat model of unilateral renal artery stenosis (n = 16) and found that the expression of renalase, a previously identified secreted amine oxidase, was markedly reduced in the ischemic compared to the non-ischemic kidney (protein: 0.295±0.085 versus 0.765±0.171, *p*<0.05). Subsequently, we utilized an isolated perfused rat kidney model to demonstrate that the clearance rate of norepinephrine decreased with reduction of perfusion flow. On the basis of these findings, we hypothesized the reduced renal blood supply which occurs in heart failure would result in impaired synthesis of renalase by the kidney and consequently reduced degradation of circulating norepinephrine. To verify this, we used a rat model of infarction-induced heart failure (n = 12 per group). In these rats, the flow velocity of renal artery, when measured at four weeks, is obviously lower in the operation group. Renal expression of renalase was reduced (protein: 0.476±0.043 for control, 0.248±0.029 for operation versus 0.636±0.151 for sham-operation) and this was associated with an increase in circulating norepinephrine (0.168±0.016 ng/mL for control, 0.203±0.019 ng/mL for operation versus 0.138±0.008 ng/mL for sham-operation).

**Conclusions/Significance:**

Renalase expression is influenced by renal blood flow and impaired synthesis of renalase by the kidney may represent a potential mechanism underlying circulating norepinephrine accumulation in heart failure.

## Introduction

Chronic heart failure occurs as a result of a variety of cardiovascular diseases, the most common being is ischemic heart disease and in particular myocardial infarction. It carries a poor prognosis, worse than that of malignant tumors collectively [1]. One of the most important pathophysiologic characteristics of heart failure is sympathetic overactivity [2], which can contribute to the progression and pathophysiology of cardiac dysfunction. The plasma concentration of catecholamines is higher in patients with heart failure than in controls [3], and the degree of elevation is inversely correlated with prognosis [4].

In the context of end-stage heart failure, levels of circulating catecholamines may be extremely high, whilst they are diminished within the tissues especially the myocardium [5]. This paradox is mainly explained by decreased tissue uptake and hence clearance of plasma norepinephrine [6]. However, the mechanism underlying this decrease in catecholamine clearance remains largely unclear. Catecholamines are mainly metabolized by the enzymes monoamine oxidase and catechol-*o*-methyl transferase. However, these two enzymes are both located intracellularly, so that they cannot degrade circulating catecholamines directly but only target them either before release from the nerve terminal or once they have been taken up by cells through one of the catecholamine uptake mechanisms.

Renalase, a renally synthesized protein mainly expressed in the glomeruli and proximal tubules, was identified as an amine oxidase in 2005 [7]. Unlike the classical amine oxidases, renalase is a secreted enzyme which can be detected in plasma and degrades circulating catecholamines. Plasma renalase is markedly reduced in chronic renal failure and nephrectomized rats, and this reduction is accompanied by a sizeable increase in circulating concentrations of catecholamines [8]. Renalase-treated animals exhibit a large reduction in blood pressure accompanied by a decreased concentration of circulating catecholamines [7].

We hypothesized that reduced blood supply to the kidney, as a result of redistribution of peripheral blood flow in heart failure, causes suppression of renalase synthesis and consequently reduced degradation of circulating catecholamines. The aims of the present study were therefore: (a) to evaluate the influence of renal blood flow on renalase synthesis using a unilateral renal artery stenosis model; (b) to ascertain the relationship between renal perfusion flow and renal metabolism of norepinephrine using an in vitro kidney perfusion model; and (c) to investigate the relationship between the expression and activity of renalase in the kidney and the plasma as well as the concentration of plasma norepinephrine in an animal model of heart failure. Our studies focused on norepinephrine rather than epinephrine, since the former appears to exert much more important effects both on the heart and vasculature than the latter, at concentrations found in the circulation both in health and in the context of heart failure.

## Methods

### Animal model of unilateral renal artery stenosis

To investigate whether renal blood flow has influence on renal synthesis of renalase, rats model of unilateral renal artery stenosis was constructed, as detailed in Methods S1.

### Norepinephrine metabolism by the kidney in vitro

To investigate whether norepinephrine can be degraded directly by the kidney, and whether perfusion flow or pressure can influence its clearance rate, we constructed isolated perfused rat kidney model as previously described [9], [10]. Full details are given in Methods S1. Norepinephrine was added to the Krebs-Henseleit bicarbonate perfusate, adjusting the concentration to 20 ng/mL.

### Animal model of acute myocardial infarction (AMI)

To evaluate the expression and activity of renalase and the concentration of plasma norepinephrine in animals with cardiac dysfunction, a rat AMI model (n = 12/group) was constructed as previously described [11], as detailed in Methods S1.

All animal research conformed to the Guide for the Care and Use of Laboratory Animals published by the US National Institutes of Health (NIH Publication No. 85-23, revised 1985) and was approved by the Ethics Review Board for Animal Studies of Nanjing Drum Tower Hospital (DTH ERBA 66.01/037D/2009).

### Renalase activity

Amine oxidase activity of renalase in the perfusate and plasma of heart failure rats was measured by Amplex Red Monoamine Oxidase Assay Kit (Invitrogen), based on the detection of H_2_O_2_ in a horseradish peroxidase-coupled reaction according to the manufacturer's instructions.

### Renal blood flow velocity

To evaluate renal blood flow of different groups in heart failure model, the renal blood flow velocity were detected using Toshiba Xario ultrasound system (SSA-680) with a 7.5 MHz linear-array transducer. The flow velocity of renal artery proximal to abdominal aorta of different groups were evaluated by a technician blinded to the grouping.

### Echocardiography and hemodynamic assessment

To evaluate cardiac function of rats post-AMI, we performed echocardiography and hemodynamic assessment four weeks after LAD ligation. Full details of the methodology are given in Methods S1.

### Plasma and tissue sample preparation

Full details are given in Methods S1.

### Plasma brain natriuretic peptide (BNP) quantification

We measured plasma BNP concentration using a commercially available kit (Uscn Life Science Inc. Wuhan, China)following the manufacturer's instructions.

### Renalase protein expression in the kidney

Renalase protein expression in the kidney was quantified by western blotting, and alpha-tubulin was also quantified in the same samples as control. Full details of the methodology are given in Methods S1. Results were presented as density values of renalase as a ratio to alpha-tubulin.

### Norepinephrine quantitation by EIA

Plasma, perfusate and urinary norepinephrine concentration were determined by competitive ELISA using a commercially available kit (Norepinephrine EIA, ALPCO), according to the manufacturer's instructions.

### Determination of plasma and perfusate renalase concentration

Plasma and perfusate renalase concentration was determined by ELISA analysis, full details are given in Methods S1.

### Immunohistochemical analysis of renalase expression in the kidney

We carried out immunohistochemical analysis to determine renalase expression and spatial distribution in the kidney. 5um paraffin-embedded kidney slices were incubated with anti-renalase antibody (1∶300, Abcam) at 4°C overnight, and visualized using EnVision detection system with peroxidase/DAB (DAKO). Renalase expression was quantified using Image Pro Plus 6.0 software (Media Cybernetics).

### Statistical analysis

All results are expressed as mean ± SEM. Comparisons between two groups (ischemia and nonischemia in renal artery stenosis model) were performed by Wilcoxon rank sum test, while comparisons of data among three groups (operation, sham-operation and control in heart failure model) were performed by Kruskal-Walls test. All data were analysed using SPSS 13.0 software (SPSS, Inc, Chicago, IL, USA). Statistical significance was defined as *P*<0.05 (two-tailed).

The authors had full access to the data and take responsibility for its integrity. All authors have read and agree to the manuscript as written.

## Results

### Protein expression of renalase in renal ischemia model

**Figure 1:**
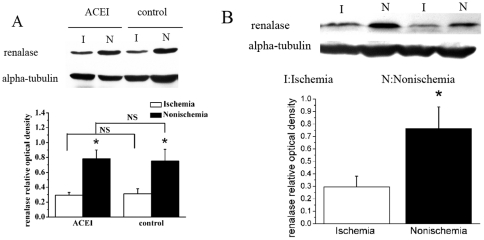
Protein expression of renalase diminished in the ischemic compared to the non-ischemic kidney. A, There is no statistic difference between renalase expression between the ACEI and control groups both in the ischemia kidney and the nonischemia one. B, Western blotting showing renalase protein expression of ischemia kidney is lower than that of the nonischemia one. Alpha-tubulin also shown as a housekeeping control. Corresponding graph shows accumulated results from n = 8 (A) or n = 16 (B) for each group (0.295±0.085 versus 0.765±0.171). *p<0.01 vs. ischemic kidney. NS: No statistical difference is existed between the two groups.

No difference was found in renalase protein expression between rats treated with angiotensin converting-enzyme inhibitor (ACEI) and those treated with corresponding vehicle (n = 8/group; Figure 1a), thus effectively excluding the possibility that renin-angiotensin-aldosterone system activity could potentially influence renalase expression. On the other hand, as shown in Figure 1b, protein expression levels was lower in the ischemic than the non-ischemic kidney, indicating that renal blood flow has an important influence on renal synthesis of renalase.

### Catecholamine degradation by the isolated perfused kidney

**Figure 2:**
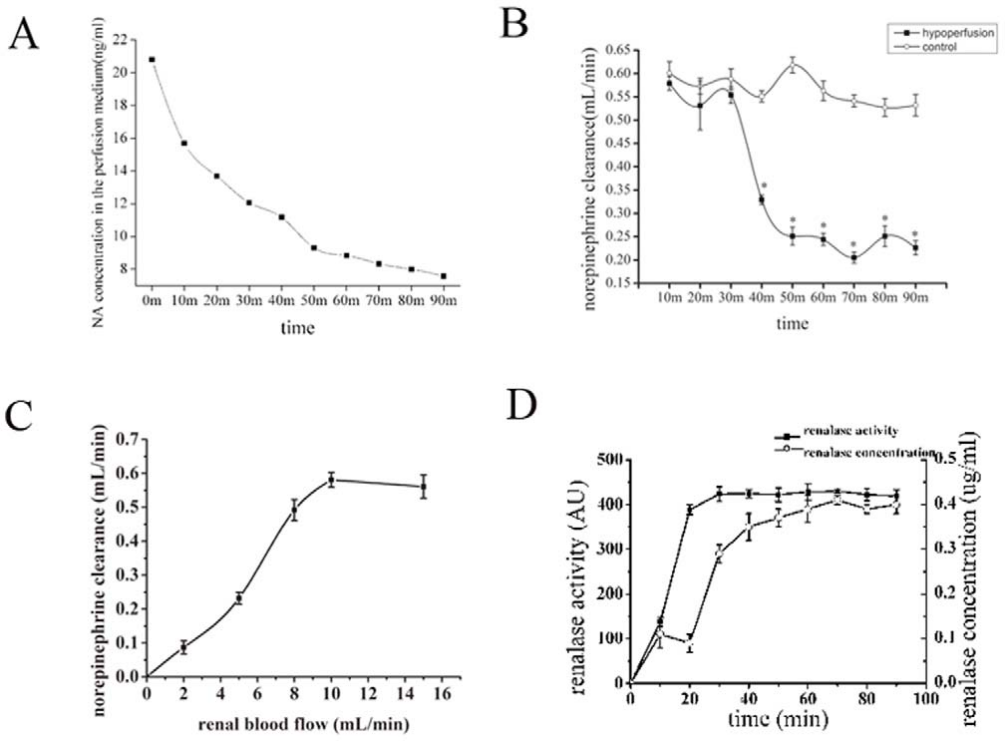
Norepinephrine clearance is decreased in the hypoperfused kidney. **A**, Concentration of norepinephrine at different time points in the perfusion medium, using a perfusion flow rate of 10 mL/min. **B**, Clearance rate of norepinephrine at conditions of different perfusion flow. In the hypoperfusion group, the perfusion flow rate is 10 mL/min for the initial 30 min, followed by 5 mL/min for the succeeding 60 min. In the control group, the perfusion flow rate is constant at 10 mL/min for 90 min. *P<0.01 vs. control. **C**, Dose-response curve analysis (renal flow/norepinephrine clearance) in isolated perfused rat kidney model. The norepinephrine clearance is increased with increment in perfusion flow below 10mL/min while the clearance remained stable at 15mL/min as compared with 10mL/min. **D**, Renalase activity and concentration in the perfusate at different time points during the perfusion. The activity and concentration are undetectable at time 0. But within 10 minutes of the initiation of norepinephrine perfusion, renalase activity and concentration increased significantly and remained high after 20 minutes during the perfusion procedure. n = 5 per group.

The concentration of norepinephrine in the perfusion medium decreased gradually during perfusion (Figure 2A). Moreover, the clearance rate of norepinephrine decreased with reduction of either perfusion flow or pressure (Figure 2B). As shown in Figure 2C, dose-response curve analysis (renal flow/norepinephrine clearance) were performed. The norepinephrine clearance is increased with increment in perfusion flow below 10mL/min while the clearance remained stable at 15mL/min as compared with 10mL/min. We also obtained measurements of creatinine clearance at the same time, and these confirmed that the isolated kidney exhibited good function in every case (data not shown). The data suggest that norepinephrine can be degraded directly by the kidney, and that perfusion flow or pressure are important factors influencing its clearance rate.

### Renalase activity

As shown in Figure 2D, renalase activity in the perfusate is undetectable at time 0. But within 10 minutes of the initiation of norepinephrine perfusion, renalase activity increased significantly and remained high after 20 minutes during the perfusion procedure. Unfortunately, we can not find the direct correlation between norepinephrine clearance and renalase activity in our experiment. Meanwhile, we also noticed that the elevation of renalase activity in the initial 20 minutes is most likely due to the increased renalase concentration in the perfusate as a result of renalase secretion by the kidney in this time course. In heart failure model, the plasma renalase activity is *91±8AU in operation group, 46±7AU in sham-operation group while 35±7AU in control group. (*p<0.05 vs. sham-operation, *p*<0.05 vs. control.)

#### Perfusate renalase concentration

As shown in figure 2D, renalase concentration in the perfusate is undetectable at time 0 while after the initiation of norepinephrine perfusion, it rose significantly and remained at a relative high level 20 minutes post-perfusion.

### Renal blood flow velocity

**Figure 3:**
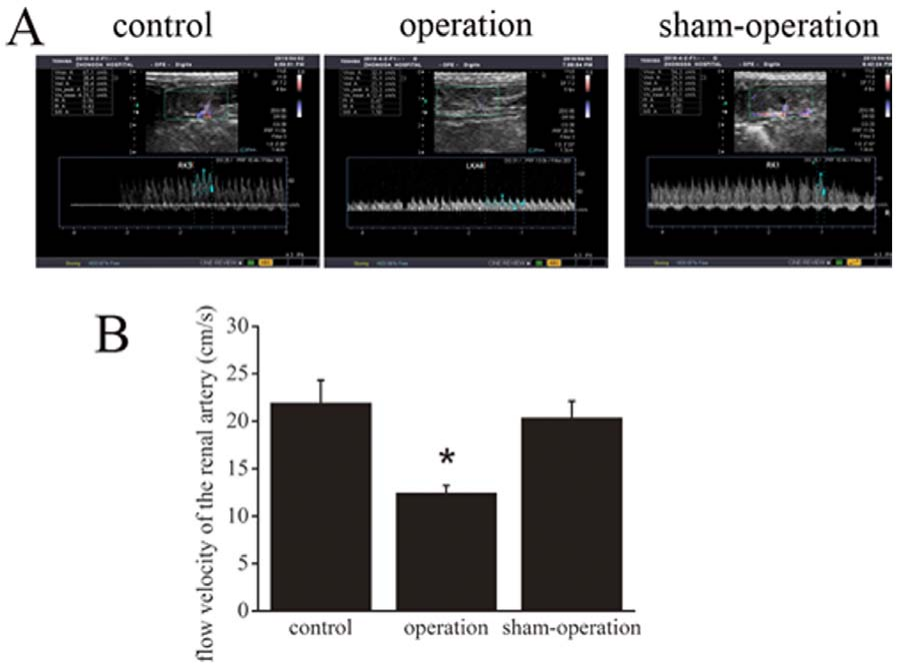
The flow velocity of renal artery is obviously lower in the operation group of heart failure model. **A**, Ultrasonic image**s** of renal artery flow velocity of different groups. **B**, Corresponding graph shows accumulated results of flow velocity from n = 12 for each group (21.93±2.40 cm/s for control, 12.43±0.82 cm/s for operation versus 20.35±1.76 cm/s for sham-operation). *p<0.05 vs. sham-operation, *p*<0.05 vs. control.

As shown in Figure 3, the flow velocity of renal artery is obviously lower in the operation group as compared to both control and sham-operation groups in heart failure model (21.93±2.40 cm/s for control, 12.43±0.82 cm/s for operation versus 20.35±1.76 cm/s for sham-operation).

### Physical characteristics and cardiac function

Heart weight/body weight (‰) and plasma BNP concentration were both higher in operation group compared to the other two groups, indicating progressive ventricular remodeling and elevated cardiac loading. As expected, systolic function, evaluated by %FS and ±dp/dt, exhibited a marked deterioration in the operation group compared with the sham operation and control groups (Table 1). Heart rate was slightly higher in the operation group, consistent with heightened sympathetic activity, although this rise did not reach statistical significance. Additionally, LVESD, LVEDD and LVEDP were both higher in operation animals compared to the other two groups, while no differences were seen among the three groups in IVSd or LVPWT (data not shown).

**Table 1:** Physical characteristics and cardiac function evaluation of rats 4 weeks post-LAD ligation.

	heart weight/body weight (‰)	BNP (pg/mL)	LVEDD (cm)	LVESD (cm)	FS (%)	HR (bpm)	LVEDP (mmHg)	±dp/dt_max_ (mmHg/s)
Operation	3.71±0.41	83.17±12.46	0.712±0.082	0.539±0.066	23.380±2.941	225.546±5.993	23.42±2.16	±(834.56±12.33)
Sham-operation	2.93±0.20*	17.39±3.13**	0.581±0.078*	0.348±0.046*	39.890±1.616*	208.800±8.576	5.82±1.27*	±(2310.45±21.58)*
Control	2.89±0.23*	16.29±2.96**	0.510±0.030**	0.293±0.021**	42.430±1.512**	210.290±6.059	3.21±1.14*	±(2834.27±19.35)*

*p<0.05 vs. operation,

**p<0.01 vs. operation.

### Protein expression of renalase in heart failure rats

**Figure 4:**
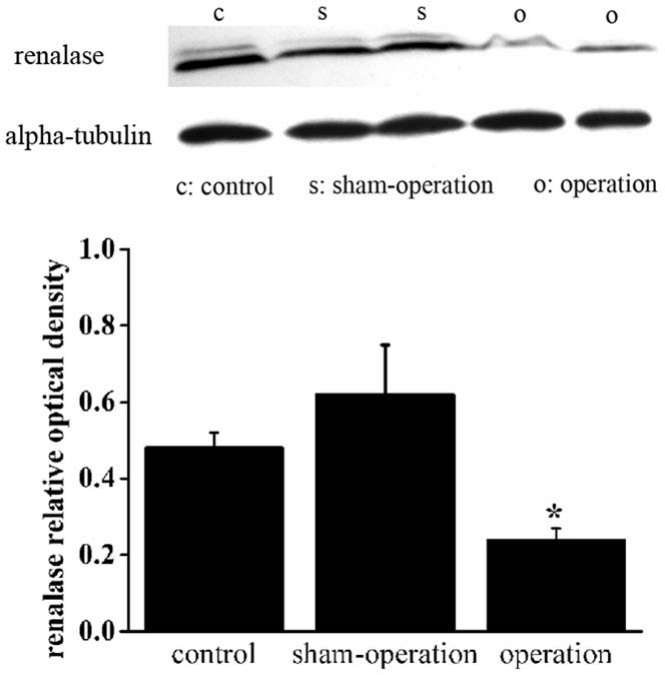
Protein expression of renalase is decreased in kidneys from heart failure rats four weeks after LAD ligation. Alpha-tubulin also shown as a housekeeping control. Corresponding graph shows accumulated results from n = 12 for each group (0.476±0.043 for control, 0.248±0.029 for operation versus 0.636±0.151 for sham-operation). *p<0.01 vs. sham-operation, *p*<0.01 vs. control.

Renalase expression decreased at the protein level in the kidneys of heart failure rats compared with those of control rats (Figure 4).

**Figure 5:**
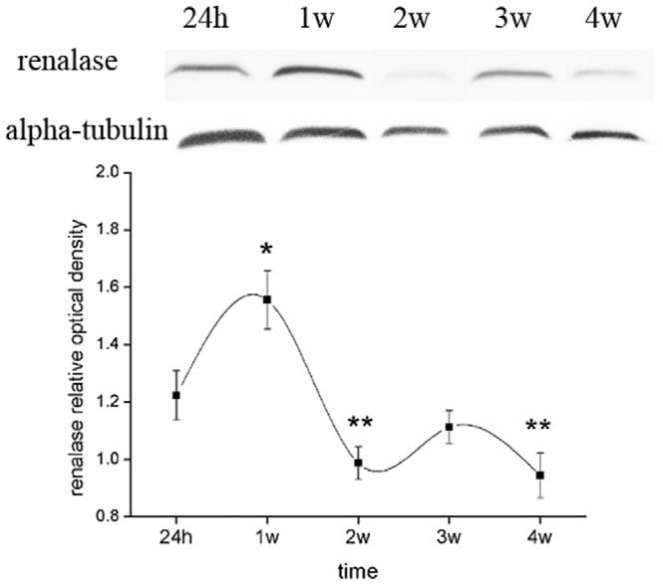
Renalase expression in the kidney increases and subsequently decreases with progression of heart failure post-LAD ligation. Western blot showing expression of renalase at different time points (24 h–4 weeks) post-LAD ligation. Alpha-tubulin expression is also shown as a housekeeping control. Corresponding graph shows accumulated results from n = 4 for each time point (1.223±0.086 for 24h, 1.556±0.102 for 1w, 0.987±0.056 for 2w, 1.112±0.057 for 3w, 0.945±0.078 for 4w). * *p*<0.05 vs. 24h, 2w, 3w and 4w. ** *p*<0.05 vs. 24h.

To determine the time course of changes in renalase expression in the kidneys, this was determined by western blotting at different time points (24 h–4 weeks) following LAD ligation. Renal renalase expression increased and reached a peak at one week post-LAD ligation, gradually decreasing thereafter (Figure 5).

### Plasma norepinephrine and renalase concentrations

**Figure 6:**
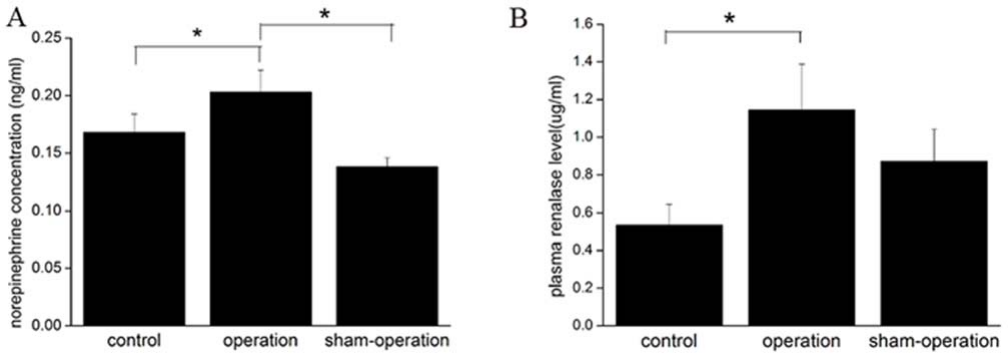
Plasma norepinephrine and renalase concentrations are both increased in heart failure rats. **A**, Plasma norepinephrine concentration in different groups (0.168±0.016 ng/mL for control, 0.203±0.019 ng/mL for operation versus 0.138±0.008 ng/mL for sham-operation). **B**, Plasma renalase concentration in different groups (0.535±0.109 ug/mL for control, 1.144±0.243 ug/mL for operation versus 0.872±0.171 ug/mL for sham-operation). n = 12 per group. *p<0.05.

Plasma norepinephrine was higher in heart failure rats than the other two groups (Figure 6A), confirming sympathetic overactivity and catecholamine accumulation as a result of heart failure. Plasma renalase was elevated in operation as compared to both sham operation and control rats (Figure 6B). We also analyzed the correlation between renalase expression in kidney and plasma levels of norepinephrine (r = −0.485, *p* = 0.110) or the correlation between plasma concentration of renalase and norepinephrine (r = −0.532, *p* = 0.075) at 4 weeks following LAD ligation, although which showed no statistical significance, the tendency of the higher renalase, the lower norepinephrine did exist in heart failure rats (data not shown).

### Pathologic analysis of renalase expression in the kidney

**Figure 7:**
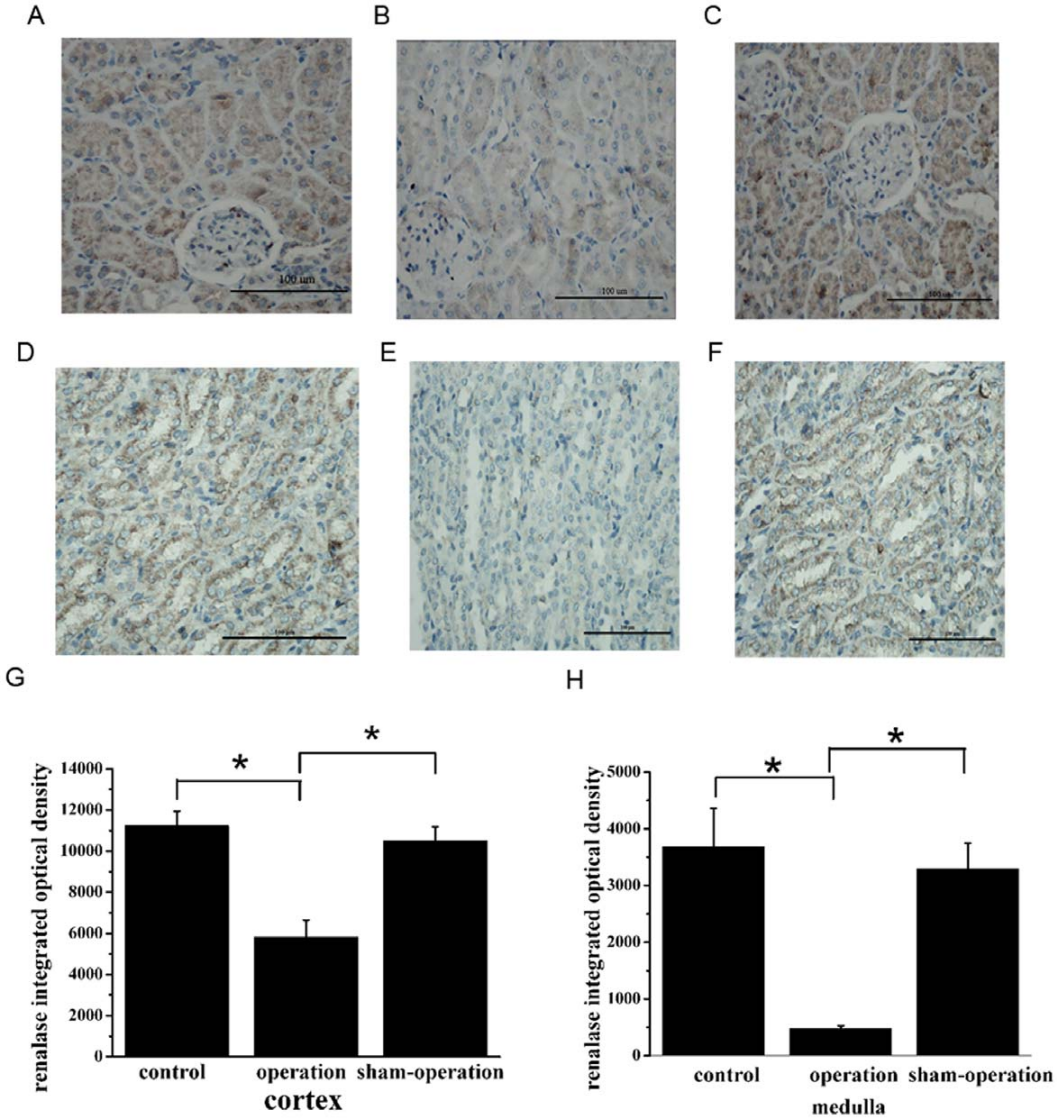
Renalase expression is reduced in the kidneys of heart failure rats. Renalase expression is demonstrated immunohistochemically in kidney cortex (**A–C**) and medulla (**D–F**). **A**, **D**, Control. **B**, **E**, Operation. **C**, **F**, Sham-operation. **G–H**, Quantification of renalase expression in different groups in renal cortex (**G**) and medulla (**H**). n = 12 per group. *p<0.01. Scale bars: 100 µm(A–F).

The spatial distribution of renalase within the kidney was mainly in proximal tubules, with much weaker expression in glomeruli. Quantitative analysis showed a reduction of renalase expression in the kidneys of heart failure rats (Figure 7A–7H), consistent with the results of protein expression as determined by western blotting.

## Discussion

Sympathetic neurohormonal overactivity and consequent accumulation of circulating catecholamines are characteristically found in heart failure. Sustained sympathetic activation leads to further deterioration of cardiac function through a variety of mechanisms including stimulation of cardiomyocyte apoptosis [12], direct toxicity to cardiomyocytes [13], and induction of ventricular dysrhythmias [14]. In the present study, we tried to explore the mechanism underlying circulating norepinephrine accumulation in heart failure with the finding that renalase protein expression was impaired in the ischemia kidney as compared to the non-ischemia one. In the isolated perfused kidney model, we found that the norepinephrine can be degraded directly by the kidney and as blood flow or pressure declined, the rate of clearance of norepinephrine decreased in parallel. In the kidneys of rats with ischemia-induced heart failure, we found that the flow velocity of renal artery and renalase protein expression were reduced with a concomitant increased concentration of circulating norepinephrine.

The catecholamine accumulation which occurs in this context may in part be explained by desensitization of arterial baroreflexes [15] and cardiopulmonary reflexes [16], as well as elevated central and circulating levels of angiotensin II [17]–[20]. However, this is unlikely to be the full explanation since, with increasing sympathetic activation, norepinephrine becomes depleted in the nerve terminals. We considered it likely, therefore, that norepinephrine metabolism is also decreased in heart failure. The present results show that, indeed, heart failure is associated with a decrease in renalase expression by the kidney, which in turn causes a decrease in norpeinephrine degradation and hence its accumulation in the circulation.

Unlike MAO-A and MAO-B, renalase is secreted into the blood and metabolizes catecholamines in the circulation [7]. Moreover, circulating renalase is largely inactive under basal conditions (in the form of prorenalase), while excess catecholamines not only stimulate its activity (through conversion to renalase) but also upregulate its synthesis and secretion [8]. Two single nucleotide polymorphisms (SNPs) of the renalase gene (rs2576178 and rs2296545) have been shown to be associated with essential hypertension in a north Han Chinese population [21]. Renalase protein expression is decreased in the hearts of neonatally nephrectomized rats compared to those of control animals, and this has been postulated to contribute to the increase in plasma norepinephrine in these rats [22].

In the present study, we found in a unilateral renal artery stenosis rat model that the synthesis of renalase by the ischemic kidney was impaired compared to the non-ischemic kidney, suggesting that renal blood flow has an important influence on renalase synthesis by the kidney. This may to a large extent explain the circulating renalase deficiency seen in patients with end-stage kidney disease. In the kidneys of rats with experimentally-induced heart failure, we found that the flow velocity of renal artery is obviously lower. Unfortunately, we can not obtain the exact data of diameter of renal artery or that of blood flow in view of the limitation of the resolution of our measurement instrument. But considering the similar renal artery diameter of rats of the same age, we believe that the renal flow velocity could at least in part represent the situation of renal blood flow. That is to say, the renal blood flow is lower in the operation group compared to the other two groups. Renalase protein expression was reduced with a concomitant increased concentration of circulating norepinephrine in our post-MI heart failure model.

It has previously been demonstrated that plasma renalase is inactive whilst urinary renalase has amine oxidase activity under basal conditions [8]. This, coupled with the finding that catecholamines can activate renalase [8], suggests two possibilities: either renalase does not exert a physiologically important function or catecholamines can be metabolized by renalase in the kidney rather than by circulating renalase, under basal conditions in vivo. To distinguish between these two possibilities, we investigated norepinephrine clearance in the isolated kidney perfusion model. Our results strongly support the notion that norepinephrine is directly degraded by the kidney. Importantly we found that, as blood flow or pressure declined, the rate of clearance of norepinephrine decreased in parallel. But as there is no clear evidence that this rapid effect is due to lack of renalase, further investigation on possible mechanism of such a rapid effect is needed.

Interestingly, the plasma renalase concentration was higher in heart failure rats compared with control animals. In the same way as sympathoadrenal activity changes from compensation in the early phase to decompensation in the late phase of heart failure, we hypothesized that renalase expression in vivo goes through similar changes as the function of heart failure progression. To confirm our hypothesis, immunoblotting analysis of renalase expression in the kidney was undertaken at different time points following LAD ligation. Intriguingly, we found that renalase protein level increased to a peak at one week, declining thereafter to reach sub-basal levels. It has previously been demonstrated that circulating catecholamines are elevated [3] whilst renal blood flow is decreased, in patients with asymptomatic left ventricular dysfunction [23]. We therefore propose that the kidney may synthesize and secrete more renalase to compensate for the increase in catecholamine concentration, during the early stage of deterioration of cardiac function and as cardiac function continues to deteriorate, renal blood flow falls to a critical level, so that renalase expression by the kidney fails to keep up with the increase in catecholamines, resulting in a phase of decompensation. However, the mechanism underlying renalase deficiency in under-perfused kidney remains largely unknown. Both anoxia and renal cell insult could be the reason, which needs further investigation.

Also, we found that the concentration and activity of plasma renalase were both higher in heart failure rats so that there was more activated renalase to degrade the increased circulating catecholamine in heart failure rats which is in favor of our hypothesis.

However, in the present study we do not provide direct evidence for a causal relationship between the increase in plasma norepinephrine of heart failure rats and reduced norepinephrine degradation by renalase, which needs further investigation. Nevertheless, our data indicate that renalase deficiency may represent a potential mechanism underlying catecholamine accumulation in heart failure. It remains to be seen whether renalase replacement may prove to be a promising therapy for heart failure.

## Supporting Information

Methods S1 Supplemental Methods
(0.05 MB DOC)

## References

[1] Kaye DM, Krum H (2007). Drug discovery for heart failure: a new era or the end of the pipeline?. Nat Rev Drug Discov.

[2] Negrao CE, Middlekauff HR (2008). Adaptations in autonomic function during exercise training in heart failure.. Heart Fail Rev.

[3] Mircoli L, Fedele L, Benetti M, Bolla GB, Radaelli A (2002). Preservation of the baroreceptor heart rate reflex by chemical sympathectomy in experimental heart failure.. Circulation.

[4] Kaye DM, Lefkovits J, Jennings GL, Bergin P, Broughton A (1995). Adverse Consequences of High Sympathetic Nervous Activity in the Failing Human Heart.. J Am Coll Cardiol.

[5] Liang CS (2007). Cardiac sympathetic nerve terminal function in congestive heart failure.. Acta Pharmacol Sin.

[6] Hasking GJ, Esler MD, Jennings GL, Burton D, Johns JA (1986). Norepinephrine spillover to plasma in patients with congestive heart failure: evidence of increased overall and cardiorenal sympathetic nervous activity.. Circulation.

[7] Xu J, Li G, Wang P, Velazquez H, Yao X (2005). Renalase is a novel, soluble monoamine oxidase that regulates cardiac function and blood pressure.. J Clin Invest.

[8] Li G, Xu J, Wang P, Velazquez H, Li Y (2008). Catecholamines regulate the activity, secretion, and synthesis of renalase.. Circulation.

[9] Russo LM, McKee M, Brown D (2006). Methyl-β-cyclodextrin induces vasopressin-independent apical accumulation of aquaporin-2 in the isolated, perfused rat kidney.. Am J Physiol Renal Physiol.

[10] Taft DR (2004). The isolated perfused rat kidney model: a useful tool for drug discovery and development.. Current Drug Discovery Technologies.

[11] Zhang J, Ding L, Zhao Y, Sun W, Chen B (2009). Collagen-targeting vascular endothelial growth factor improves cardiac performance after myocardial infarction.. Circulation.

[12] Communal C, Singh K, Pimentel DR, Colucci WS (1998). Norepinephrine stimulates apoptosis in adult rat ventricular myocytes by activation of the ß-adrenergic pathway.. Circulation.

[13] Mann DL, Kent RL, Parsons B, IV GC (1992). Adrenergic effects on the biology of the adult mammalian cardiocyte.. Circulation.

[14] Meredith IT, Broughton A, Jennings GL, Esler MD (1991). Evidence of a selective increase in cardiac sympathetic activity in patients with sustained ventricular arrhythmias.. N Engl J Med.

[15] Dibner-Dunlap ME, Thames MD (1989). Baroreflex control of renal sympathetic nerve activity is preserved in heart failure despite reduced arterial baroreceptor sensitivity.. Circ Res.

[16] DiBona GF, Sawin LL (1995). Increased renal nerve activity in cardiac failure: Arterial vs. cardiac baroreflex impairment.. Am J Physiol.

[17] Dibona GF, Jones SY, Brooks VL (1995). ANG II receptor blockade and arterial baroreflex regulation of renal nerve activity in cardiac failure.. Am J Physiol.

[18] Gao L, Wang W, Li YL, Schultz HD, Liu D (2004). Superoxide mediates sympathoexcitation in heart failure: Roles of angiotensin II and NAD(P)H oxidase.. Circ Res.

[19] Zucker IH, Schultz HD, Li YF, Wang Y, Wang W (2004). The origin of sympathetic outflow in heart failure: the roles of angiotensin II and nitric oxide.. Prog Biophys Mol Biol.

[20] Reid IA (1992). Interactions between ANG II, sympathetic nervous system, and baroreceptor reflexes in regulation of blood pressure.. Am J Physiol.

[21] Zhao Q, Fan Z, He J, Chen S, Li H (2007). Renalase gene is a novel susceptibility gene for essential hypertension: a two-stage association study in northern Han Chinese population.. J Mol Med.

[22] Ghosh SS, Krieg RJ, Sica DA, Wang R, Fakhry I (2009). Cardiac hypertrophy in neonatal nephrectomized rats: the role of the sympathetic nervous system.. Pediatr Nephrol.

[23] Magri P, Rao MAE, Cangianiello S, Bellizzi V, Russo R (1998). Early impairment of renal hemodynamic reserve in patients with asymptomatic heart failure is restored by angiotensin II antagonism.. Circulation.

