# Supplementary material for: Renalase Deficiency in Heart Failure Model of Rats—A Potential Mechanism Underlying Circulating Norepinephrine Accumulation
Source: PLoS One. 2011 Jan 31;6(1):e14633. doi: 10.1371/journal.pone.0014633 (PMC3031511; doi:10.1371/journal.pone.0014633)
Supplement: Methods S1 — Supplemental Methods (0.05 MB DOC) [file pone.0014633.s001.doc]

**SUPPLEMENTAL MATERIAL**

**Renalase Deficiency in Heart Failure Model of Rats—A Potential Mechanism Underlying Circulating Norepinephrine Accumulation**

Rong Gu^1^, Wen Lu^1^, Jun Xie^1^, Jian Bai^1^, Biao Xu^1^

^1^From the Department of Cardiology (R.G., W.L., J.X., J.B., B.X.), Affiliated Drum Tower Hospital, Nanjing University Medical School, Nanjing 210008, China.

**Address correspondence and reprint requests to：**

Professor Biao Xu, MD, PhD, Department of Cardiology, Affiliated Drum Tower Hospital, Nanjing University Medical School, Nanjing 210008, China. *Tel:* +86-25-831-052-05. *Fax:* +86-25-833-0 80-59. *E-mail:* [xubiao@medmail.com.cn](mailto:xubiao@medmail.com.cn)

**Supplemental Methods**

**Animal model of unilateral renal artery stenosis**

Male Sprague-Dawley rats aged 8 weeks weighing 180-200g were used. One group received perindopril 4 mg/kg/day for one week (n=8) before surgery whilst another (control) group received the corresponding volume of saline for one week (n=8). At the end of this time, plasma renin activity and the concentration of circulating angiotensin I and II were measured using commercially available radioimmunoassay kits (North Institute of Biological Technology, Beijing, China), to ensure adequate blockade of the renin-angiotensin-aldosterone system in the perindopril group. After anesthesia with ketamine and diazepam (50 mg/kg and 5 mg/kg respectively) intraperitoneally, an abdominal left para-midline incision was made and the left renal artery was separated from the vein followed by clipping of the renal artery using a silver clip with internal diameter 0.2 mm (Shanghai Alcott Biotech Co., China). The peritoneum, muscles and skin were sutured sequentially.

**Norepinephrine metabolism by the kidney in vitro**

The isolated perfused rat kidney model was constructed as previously described. Following induction of anesthesia as described above, a midline incision was performed. The major anatomical structures including the right kidney, right renal artery, right ureter and right superior mesenteric artery were identified and separated carefully. After isolation, the right renal artery was cannulated through the superior mesenteric artery and the right ureter was also cannulated; the cannulae were ligated in place. The right kidney was then removed and put into the perfusion apparatus, and perfused through the renal artery cannula with Krebs-Henseleit bicarbonate (116 mmol/L NaCl, 25 mmol/L NaHCO_3_, 3.87 mmol/L KCl, 2.44 mmol/L MgSO_4_(7H_2_O), 1.9 mmol/L CaCl_2_, 1.53 mmol/L KH_2_PO_4_, 6% BSA (Fraction V), 5.6 mmol/L glucose, 1.3 mmol/L creatinine). Norepinephrine was added to the KHS perfusate, adjusting the concentration to 20 ng/mL. In the hypoperfusion group, the perfusion flow was set at 10 mL/min for the first 30 min followed by 5 mL/min for the following 60 min, whilst in the control group the perfusion flow was kept constant at 10 mL/min for 90 min. In both groups, every 10 minutes during perfusion, 500 uL perfusate and urine were collected for subsequent analysis, and urine volume was recorded. Also, to analysis the relationship between renal flow and norepinephrine clearance, we repeated the perfusion experiment and adjust the perfusion flow at 2, 5, 8, 10 and 15 mL/min respectively. Each flow were lasted for 20 min while the samples were collected as previously described. Each experiment were repeated for 5 times. Norepinephrine concentration in the perfusion medium and urine at different time points was determined by a commercially available norepinephrine EIA kit (ALPCO), and creatinine concentration was determined chemically. The clearance of creatinine and norepinephrine (C_Cr_ and C_NE_ ) were calculated from urine volume per minute (V) and their concentration in the perfusion medium (P) and urine (U) using the standard formula:

C = U×V/P

**Animal model of acute myocardial infarction (AMI)**

Briefly, after thoracic incision in the fourth intercostal space in rats on a ventilator (Jiangxi Teli, China), the left anterior descending coronary artery (LAD) was ligated using a 6/0 silk suture 1-2 mm below the tip of the left atrial appendage. Following ligation, it was evident that there was weakening of beating of the apex cordis and anterior wall of the left ventricle, as well as a change in color from rubicund to pale. After the lung was inflated with positive end-expiratory pressure, the thorax was closed in three layers: chest cavity, muscles and skin. Myocardial ischemia was confirmed by 2,3,4-triphenyltetrazolium chloride dye (TTC; Sigma-Aldrich) staining and hematoxylin-eosin staining.

**Echocardiography and** **hemodynamic assessment**

Rats underwent transthoracic echocardiography assessment using a cardiovascular ultrasound system (SONOS model 5500, Hewlett-Packard Co.) with an 8 MHz linear-array transducer. Rats were anesthetized as outlined above and placed in a supine position on a warm blanket. Long- and short-axis two-dimensional views as well as M-mode tracings were obtained. Left ventricular end-systolic dimension (LVESD) and end-diastolic dimension (LVEDD) as well as interventricular septal thickness in diastole (IVSd) or left ventricular posterior wall thickness (LVPWT) were all measured by an observer blinded to the surgery. All measurements were repeated for at least three consecutive pulsation cycles, and the data were averaged before analysis. Percent left ventricular fractional shortening (%FS) was obtained as follows:

%FS = (LVEDD-LVESD)/LVEDD ×100 (%).

Following echocardiography assessment, a polyethylene catheter (PE 50, Becton-Dickinson) which connected to a pressure transducer (MPU-0.5, Nihon Kohden, Japan) was introduced into the left ventricular via the right carotid artery to obtain the hemodynamic indexes as LV end-diastolic pressure (LVEDP), maximum ±dP/dt and heart rate (HR). All measurements were performed by an observer blinded to treatment.

**Plasma and tissue sample preparation**

In the renal ischemia model, 24 hours after surgery, both kidneys were frozen in liquid nitrogen for western blotting analysis. In the heart failure model, after hemodynamic assessment, blood samples were collected in heparinized tubes before hearts were arrested in the diastolic phase by intravenous injection of 2 mol/L KCl. Heart weight and body weight were recorded followed by heart and one of the kidneys were fixed in 4% neural formalin for 24-48 h at room temperature, and then embedded in paraffin wax and sliced into 5 um slices for subsequent histologic analysis. The other kidney was processed as described above for western blotting.

**Renalase protein expression in the kidney**

Frozen kidneys were homogenized in lysis buffer of the following composition: Tris-HCl 25 mmol/L, NaCl 150 mmol/L , phenylmethanesulfonyl fluoride 1mmol/L, EDTA 1 mmol/L, NaF 50 mmol/L, sodium orthovanadate 1 mmol/L, Triton-X 1% (v/v) and protease inhibitor cocktail (Roche); pH 7.6. Protein concentrations were measured by BCA protein assay kit (Pierce). 100μug protein per sample was separated on a 10% SDS-polyacrylamide gel and then transferred to a polyvinylidene difluoride membrane (Millipore, Bedford, MA, USA). The membrane was blocked with 5% skimmed milk in 0.1% Tris-buffered saline-Tween (TBST) and then probed with antibodies directed against renalase (1:500, Abcam) or alpha-tubulin (1:1500, Cell Signaling Technology, Beverly, MA, USA), at 4°C overnight, followed by washing three times in TBST. The membrane was then incubated with horseradish peroxidase-conjugated anti-goat (1:5000, Dako) or anti-rabbit (1:3000, Jackson ImmunoResearch) IgG for 2h at room temperature. Bands were displayed using an enhanced chemiluminescence detection system (Pierce Biotechnology Inc, Rockford, IL, USA).

**Determination of plasma and perfusate renalase concentration**

After centrifugation at 825 ×g at 4°C for 20 min, the plasma and perfusate was collected and stored at -80°C until analysis. 96-well plates were coated with renalase peptide (Abcam) at a concentration of 0.5ug/ml, diluted in bicarbonate buffer pH 9.6 at 4°C overnight. After blocking with 1% bovine serum albumin (BSA) at room temperature for 1 hour, plasma or perfusate samples and anti-renalase antibody (1:32000, Abcam) 50 uL each were added to the coated wells and incubated at 37°C for 2 h. After washing with PBS, horseradish peroxidase (HRP)-conjugated rabbit anti-goat IgG (1:5,000, Dako) was used as secondary antibody. After the termination of coloration, OD at 450nm was measured using a microplate reader (TECAN, SUNRISE, Australia).
